# Forward Osmosis for Produced Water Treatment: Comparative Performance Evaluation of Fabricated and Commercial Membranes

**DOI:** 10.3390/polym18020197

**Published:** 2026-01-10

**Authors:** Sunith B. Madduri, Raghava R. Kommalapati

**Affiliations:** 1 Center for Energy and Environmental Sustainability, Prairie View A&M University, Prairie View, TX 77446, USA; sbmadduri@pvamu.edu; 2 Department of Civil and Environmental Engineering, Prairie View A&M University, Prairie View, TX 77446, USA

**Keywords:** cellulose triacetate (CTA) membrane, electrospun nanofibrous membrane, zwitterionic surface modification, forward osmosis: osmotic draw solutions (MgCl_2_, Na_3_PO_4_), GC-MS, produced water treatment

## Abstract

Produced water (PW) generated from oil and gas operations poses a significant environmental challenge due to its high salinity and complex organic–inorganic composition. This study evaluates forward osmosis (FO) as an energy-efficient approach for PW treatment by comparing a commercial cellulose triacetate (CTA) membrane and a fabricated electrospun nanofibrous membrane, both modified with a zwitterionic sulfobetaine methacrylate/polydopamine (SBMA/PDA) coating. Fourier Transform Infrared Spectroscopy (FTIR) spectra verified the successful incorporation of SBMA and PDA through the appearance of characteristic sulfonate, quaternary ammonium, and catechol/amine-related vibrations. Scanning electron microscopy (SEM) imaging revealed the intrinsic dense surface of the CTA membrane and the highly porous nanofibrous architecture of the electrospun membrane, with both materials showing uniform coating coverage after modification. Complementary analyses supported these observations: X-ray Photoelectron Spectroscopy (XPS) confirmed the presence of nitrogen, sulfur, and chlorine containing functionalities associated with the zwitterionic layer; Thermogravimetric Analysis (TGA) demonstrated that surface modification did not compromise the thermal stability of either membrane; and contact-angle measurements showed substantial increases in surface hydrophilicity following modification. Gas chromatography–mass spectrometry (GC–MS) analysis of the Permian Basin PW revealed a chemically complex mixture dominated by light hydrocarbons, alkylated aromatics, and heavy semi-volatile organic compounds. FO experiments using hypersaline PW demonstrated that the fabricated membrane consistently outperformed the commercial membrane under both MgCl_2_ and Na_3_PO_4_ draw conditions, achieving up to ~40% higher initial water flux and total solids rejection as high as ~62% when operated with 2.5 M Na_3_PO_4_. The improved performance is attributed to the nanofibrous architecture and zwitterionic surface chemistry, which together reduced fouling and reverse solute transport. These findings highlight the potential of engineered zwitterionic nanofibrous membranes as robust alternatives to commercial FO membranes for sustainable produced water treatment.

## 1. Introduction

The rapid expansion of petroleum extraction, particularly from unconventional shale formations, has led to unprecedented volumes of produced water (PW). Each well can generate several million gallons of liquid waste throughout its productive life, making PW one of the most significant sources of liquid waste associated with the oil and gas industry [1,2,3]. Unlike typical industrial wastewater, PW is not defined by a single major contaminant group; rather, it is an evolving and highly complex mixture influenced by geological formation characteristics, drilling chemistry, and reservoir management [4,5]. Its composition usually contains salinity levels several times greater than seawater, along with dissolved and dispersed hydrocarbons, organic residues, heavy metals, radio nuclides, suspended particulates, and numerous chemical additives used during drilling, fracturing, and well maintenance [6]. Because of this diversity, PW poses both acute and chronic toxicity risks, with potential impacts ranging from endocrine disruption and carcinogenicity to bioaccumulation in aquatic and terrestrial food chains [4,5,7].

Environmental concerns surrounding PW are substantial. Extremely high salinity and ionic strength can impair soil structure, inhibit plant growth, disrupt aquatic osmoregulation, and suppress microbial activity that underpins natural biogeochemical cycles [1,8]. Persistent organic compounds and hydrocarbons tend to accumulate in sediments and living organisms, while trace metals and radionuclides can migrate through groundwater systems or concentrate through trophic levels. Surfactants and chelating agents commonly present in PW can enhance contaminant mobility, increasing the likelihood of subsurface infiltration and long-term ecosystem exposure [9,10,11]. Consequently, improper disposal or partially treated discharge represents a significant environmental liability, prompting regulatory agencies to implement stricter discharge standards and guidelines for reuse [4,5].

At the same time, global water stress has intensified interest in the beneficial reuse of industrial water sources. Many oil-producing regions are situated in arid and semi-arid climates, where freshwater scarcity poses a persistent challenge for agriculture, municipal use, and industrial development [2,12]. When treated effectively, PW could serve as an unconventional water source for activities such as enhanced oil recovery, drilling operations, irrigation of salt-tolerant crops, industrial cooling, and, with advanced treatment, even a potable supply [13]. Reimagining PW as a recoverable resource aligns with circular water economy principles and emerging net-zero water management strategies; however, this shift requires treatment technologies that are not only practical but also energy-efficient, resilient to fouling, and economically feasible at scale [14,15].

Membrane-based desalination processes have gained attention as promising platforms for PW treatment [16]. FO offers advantages over pressure-driven systems because of its lower energy demand, reduced compaction, and tolerance to hypersaline and chemically complex feedwaters [17,18]. Recent advances in high-pressure reverse osmosis (HPRO) have demonstrated its capability to desalinate hypersaline brines at operating pressures exceeding 120 bar, making it a viable option for direct treatment of highly saline produced waters [19]. However, severe membrane compaction and performance degradation under sustained ultra-high pressures remain significant challenges, particularly for chemically complex feeds. In contrast, FO operates without applied hydraulic pressure, avoiding compaction while offering improved tolerance to hypersaline and organic-rich matrices, and is therefore best positioned as a complementary or pretreatment process rather than a replacement for pressure-driven desalination technologies. However, conventional cellulose triacetate (CTA) FO membranes struggle with internal concentration polarization, severe organic fouling, and irreversible loss of permeability when exposed to authentic PW [20,21]. These limitations have motivated the development of membranes with engineered surface chemistries and tailored morphologies to improve wettability, antifouling behavior, and long-term durability. Electrospun nanofibrous substrates offer a versatile foundation for such enhancements due to their interconnected pore structure, adjustable fiber diameter, and significant functional surface area, which is suitable for post-fabrication chemical modification [22,23].

A wide range of strategies have been explored to improve membrane performance, including PEG-based hydrophilic grafting, polyelectrolyte layer-by-layer coatings for charge regulation, and the incorporation of nanomaterials such as graphene, titanium dioxide, or silver to introduce photocatalytic or antimicrobial functionality [24,25]. While these approaches have yielded benefits, challenges remain regarding coating durability, leaching risks, long-term hydration stability, and sensitivity to extreme salinity conditions [26,27,28,29].

More recently, zwitterionic coatings have emerged as a promising antifouling solution due to their ability to form strong hydration layers that inhibit foulant attachment through steric and charge-neutral repulsion [30,31]. Sulfobetaine based chemistries have demonstrated superior hydration capacity and resistance to hydrophobic, proteinaceous, and biologically derived fouling agents compared to PEG or conventional charged coatings [32,33]. Although surface modification of commercially available FO membranes has been widely used to improve wettability and fouling resistance, these approaches primarily alter the active-layer surface chemistry while the internal support-layer architecture remains essentially fixed when off-the-shelf membranes are employed. In practice, the pore connectivity, tortuosity, thickness, and structural parameter governing internal concentration polarization are dictated by manufacturer design choices and cannot be systematically varied by the end user within a comparative study. While phase inversion can indeed tune porosity and cross-sectional morphology during membrane fabrication, such manufacturing-level control is not accessible when commercial membranes are used as received [16,34]. Separately, electrospun nanofibrous membranes have been investigated for FO applications due to their high porosity and interconnected pore structure; however, many reported systems lack advanced surface chemistries capable of resisting the complex organic and inorganic foulants present in produced water [35]. As a result, key challenges such as internal concentration polarization, organic fouling by hydrocarbons, and reverse solute transport often persist [36,37].

The integration of an electrospun nanofibrous substrate with a zwitterionic surface coating represents a promising yet underexplored strategy to address these challenges simultaneously [35,38]. The nanofibrous architecture offers reduced mass-transfer resistance and enhanced water permeability, while zwitterionic functional groups form stable hydration layers that suppress foulant adhesion and solute back-diffusion [29,39,40]. This combined design approach is particularly relevant for hypersaline produced water treatment, where extreme salinity and complex organic mixtures demand membranes that couple high permeability with robust antifouling performance. In this work, a dual-function surface modification protocol employing sulfobetaine methacrylate (SBMA) and polydopamine (PDA) was applied to both commercial CTA forward osmosis membranes and fabricated PEI-based electrospun nanofibrous substrates. PDA served as an intermediate adhesive layer formed under mild alkaline conditions, enabling stable SBMA attachment and long-term coating durability. The resulting membranes were systematically evaluated in terms of their structural, chemical, and separation performance under realistic operating conditions for produced water.

## 2. Materials and Methods

### 2.1. Chemicals, Reagents, and Feedwater Sources

All reagents used in this study were of analytical grade and were used without further purification. The polymer system employed for electrospinning consisted of polyetherimide (PEI) purchased from Sigma Aldrich (St Louis, MO, USA) as the primary polymer, while N-methyl-2-pyrrolidone (NMP) purchased from Thermo fisher scientific (Wardhill, MA, USA), and N, N-dimethylformamide (DMF) purchased from Thermo fisher scientific (Fairlawn, NJ, USA), served as the mixed solvent system. Lithium chloride (LiCl_2_) purchased from Thermo fisher scientific (Fairlawn, NJ, USA), was used as a conductivity and spinnability enhancer. Reduced graphene oxide (rGO) purchased from ACS materials (Dayton, OH, USA), was added as a nanofiller at an optimized loading level to enhance hydrophilicity and mechanical integrity. For surface functionalization, sulfobetaine methacrylate (SBMA), and dopamine hydrochloride purchased from Sigma Aldrich (St Louis, MO, USA) were used as zwitterionic precursors, while Trizma-HCl buffer purchased from Sigma Aldrich (St Louis, MO, USA) was used to adjust the coating solution to a mildly alkaline pH. Ultrapure water was used for coating preparation, membrane rinsing, and characterization procedures. A commercial cellulose triacetate FO membrane (Sterlitech FO-CF042) was included as the control membrane. Two different salts were used as draw-solution components in FO trials: magnesium chloride (MgCl_2_) at 2.5 M and 3.5 M was purchased from Thermo fisher scientific (Fairlawn, NJ, USA), and trisodium phosphate (Na_3_PO_4_) at 1.5 M and 2.5 M concentrations were purchased from Fischer bioreagents (Fairlawn, NJ, USA). PW was collected from an active oil-field separation facility in sealed, opaque, high-density polyethylene containers, and stored at 4 °C upon arrival. PW was meticulously filtered through Whatman filter paper with a pore size of 1.4 µm to effectively eliminate any undispersed particles or aggregates, ensuring the highest quality and performance for FO applications. Before FO testing, PW was analyzed for conductivity, pH, ionic strength, and total solids (TS≈) at approximately 135 g/L to establish a baseline composition.

### 2.2. Fabrication of Electrospun Nanofibrous Substrates

Electrospun nanofibrous substrate membranes were prepared using a mixed-solvent polymer system following the electrospinning methodology previously established in our publication [35]. In brief, electrospinning was carried out at an applied voltage of approximately 24–25 kV using a solution flow rate of 0.8–1.0 mL h^−1^ and a needle-to-collector distance of 15 cm. The polymer solution was prepared, conditioned, and electrospun under controlled environmental and operational conditions to ensure the development of a uniform nanofibrous mat with an interconnected pore structure suitable for FO applications. The electrospun membranes were collected on a rotating drum collector, dried to remove residual solvent, and then preserved in a desiccator prior to the surface modification stage. All process parameters including polymer concentration, solvent ratios, nanomaterial loading, electrospinning voltage, feed rate, and collection distance were consistent with the referenced study to maintain material reproducibility and performance comparability.

### 2.3. Zwitterionic Surface Functionalization of Commercial and Electrospun Membranes

To ensure direct performance comparison, both the commercial CTA membrane and the electrospun nanofibrous substrates underwent the same surface functionalization procedure. A zwitterionic coating solution was freshly prepared by dissolving 5 g of SBMA and 1.5 g of dopamine hydrochloride in 100 mL of ultrapure water, followed by the addition of 615 mg of Trizma-HCl to adjust the working pH to approximately 8.5, which is required to initiate dopamine oxidation under mild alkaline conditions [35]. Each membrane sheet was carefully placed on a flat support with its active surface exposed, and the coating mixture was gently poured over the surface to allow complete wetting. During the coating reaction, the membranes were placed on a rocking platform shaker, which helped maintain uniform oxygen exposure and promoted in situ oxidative polymerization of dopamine, while simultaneously anchoring SBMA to the PDA network. At the end of the coating period, the membranes were thoroughly rinsed to remove any loosely bound material and then placed in ultrapure water overnight, allowing for the complete detachment of any unreacted or weakly adhered molecules. All modified membranes were dried at room temperature and stored in sealed, contamination free containers until characterization and FO testing.

### 2.4. Characterization

A series of analytical techniques were employed to assess the impact of fabrication and zwitterionic modification on membrane structure, chemistry, and surface properties. FTIR spectroscopy was performed to verify the successful incorporation of PDA and SBMA through the presence of characteristic functional groups (Nicolet Summit, Thermo Fisher, West Palm Beach, FL, USA). SEM imaging was used to observe the surface morphology, coating consistency, and any visible fouling layer that formed after FO operation (FE-SEM) (JSM-6010LA, JEOL, Tokyo, Japan). XPS analysis was conducted to further confirm elemental composition and changes in chemical bonding at the modified membrane surface. To determine stability under thermal conditions, TGA was performed (PerkinElmer, Waltham, MA, USA). Static water-contact-angle measurements were used as an indicator of surface wettability and hydrophilicity improvement following zwitterionic treatment. Additionally, physical condition, and visible structural integrity were recorded before testing. GC–MS analysis was performed to analyze the PW matrix, which revealed a chemically complex mixture dominated by light hydrocarbons, alkylated aromatics, and heavy semi-volatile organic compounds (Shimadzu QP-2020, Tokyo, Japan).

### 2.5. Forward Osmosis Operation

Forward osmosis experiments were conducted using a laboratory-scale crossflow FO unit (Sterlitech CF042 FO cell, Sterlitech Corporation, Auburn, WA, USA) operated predominantly in an active layer-facing feed (AL-FF) configuration. The effective membrane area exposed to flow was 42 cm^2^. Both the feed and draw solutions were circulated through rectangular flow channels with a width of 2.0 cm and a channel height of 0.2 cm. Two osmotic agents were investigated as draw solutions: MgCl_2_ and Na_3_PO_4_, which were prepared using analytical grade reagents dissolved in ultrapure water to the required molarity. MgCl_2_ solutions were used mainly due to their high osmotic pressure and low cost. Na_3_PO_4_ was examined as an alternative high-driving-force salt, owing to its multivalent anionic species and potential for lower reverse solute permeation. During FO testing, temperature, crossflow velocity, and hydraulic conditions were held constantly. Feed and draw reservoirs were continuously circulated to maintain uniform concentration. Water flux for all FO experiments was determined gravimetrically using a Mettler Toledo analytical balance, which continuously recorded the mass increase in the draw solution reservoir as a function of time. The crossflow velocity was maintained constant for both commercial CTA and the fabricated electrospun membranes, with the feed and draw streams operating at a flow rate of 0.75 L min^−1^ optimized PW treatment conditions. Water flux (Jw) was the volume of water permeate through the unit surface area of the membrane per unit time. Total solids (TS) rejection was assessed from the change in feed conductivity using Equation (2). The osmotic driving force across the membrane was estimated using the van’t Hoff relation (Equation (3)), incorporating the osmotic coefficient and the effective van’t Hoff factor (i) of the dissolved draw solutes (MgCl_2_ and Na_3_PO_4_). Flux evolution, fouling-induced decline, and solute leakage behavior were monitored throughout each test to evaluate the stability and comparative performance of commercial and fabricated membranes during PW filtration [16,35].

(1) $$J_{w}=\frac{\Delta m}{\rho A\Delta t}$$

(2) $$\%R=(\frac{Cr}{Cf}-1)\times 100$$

(3) π=iMRT

where J_w_ = water flux (LMH), Δm = mass change (kg), ρ = density of water (kg L^−1^), Δt = Time (hours), A = membrane area (m^2^), C_f_, C_r_ = feed and retentate concentrations, i = van’t Hoff factor (e.g., MgCl_2_ ~3; Na_3_PO_4_ ~4), M = molarity of draw solute, R = universal gas constant, T = absolute temperature.

## 3. Results

### 3.1. Characterization

#### 3.1.1. FTIR

**Figure 1:**
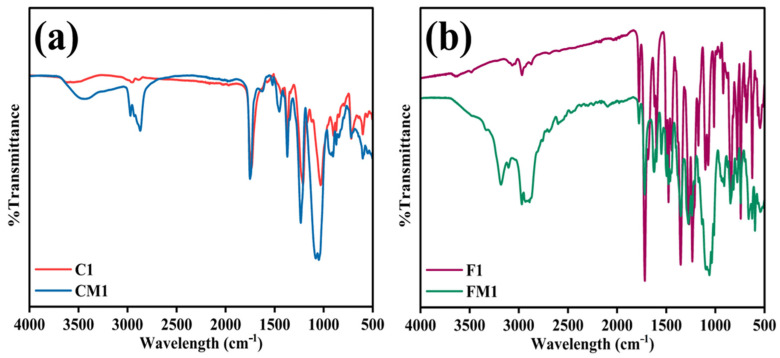
FTIR Spectra of (**a**) Commercial raw and modified membrane; (**b**) Fabricated raw and modified membrane.

The FTIR spectrum presented in Figure 1a,b was evaluated to verify the successful attachment of the zwitterionic coating onto both the pristine commercial (C1) and pristine fabricated (F1) membranes. The modified membranes (CM1 and FM1) exhibit distinct changes in their spectral profiles compared to their unmodified membranes, indicating the incorporation of SBMA and PDA functional groups on the membrane surface. The FTIR spectrum C1 shows characteristic peaks that are expected for a cellulose triacetate-based material. Typical absorption bands observed include a broad and moderate peak in the region ~3500–3200 cm^−1^, generally associated with O–H stretching vibrations due to residual surface hydroxyl groups or absorbed moisture. Pronounced peaks near ~1740–1720 cm^−1^, attributed to C=O stretching of ester linkages, which confirms the triacetate functional groups [41]. Multiple peaks observed in the 1200–1000 cm^−1^ range, corresponding to C–O–C and C–O stretching vibrations present in the acetate and polysaccharide backbone. Following zwitterionic modification (CM1), noticeable alterations appear across the spectral range (Figure 1a). The reduction in relative transmittance near ~3300 cm^−1^ suggests an increase in surface hydrophilicity arising from SBMA quaternary ammonium and sulfonate hydration groups, as well as PDA catechol and amine functionalities [42]. A slight broadening or shift is observed near the amide/imine region (~1650–1580 cm^−1^), which is consistent with C=N or N–H bending vibrations, confirming the deposition of PDA [43]. Additional subtle changes in the fingerprint region (~1200–900 cm^−1^) may be related to sulfonate (–SO_3_^−^) and quaternary ammonium (–N^+^(CH_3_)_2_) groups contributed by SBMA [44]. The observed modifications collectively demonstrate that the coating layer has adhered successfully to the commercial membrane surface. The pristine electrospun membrane (F1) exhibits a distinctly different spectral profile due to its nanofibrous polymer composition. The broad absorption in the ~3500–3300 cm^−1^ region corresponds to N–H/O–H stretching, while additional features between ~1650–1550 cm^−1^ can be attributed to amide-type or aromatic ring-related vibrations, especially when rGO is incorporated into the polymer matrix. Peaks appearing around ~1200–1000 cm^−1^ reflect the presence of polymeric backbone C–O, C–N, or C–O–C functional groups. The modified fabricated membrane (FM1) exhibits stronger and broader absorbance in the ~3400–3200 cm^−1^ region compared to the unmodified membrane, consistent with enhanced hydrogen bonding and surface hydration, which is characteristic of zwitterionic surfaces (Figure 1b). The distinct emergence or intensification of peaks near ~1650–1580 cm^−1^ is associated with PDA-related indole or catechol aromatic structures and secondary amine vibrations. Moreover, the appearance or increased intensity of signals near ~1040–1030 cm^−1^ can be correlated with the sulfonate (–SO_3_^−^) symmetric stretching mode originating from SBMA, confirming its presence on the modified nanofiber surface [41,42,43,44].

#### 3.1.2. TGA

**Figure 2:**
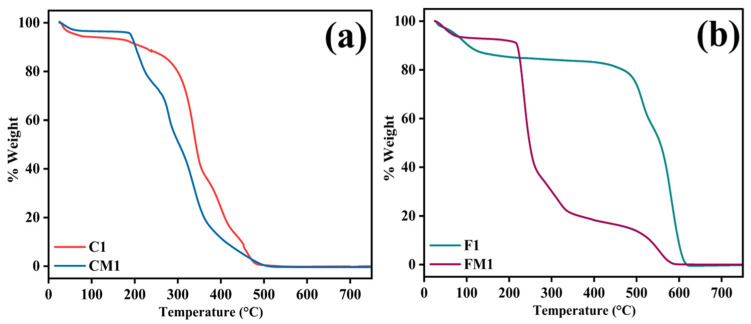
TGA graphs of (**a**) Commercial raw and modified membrane; (**b**) Fabricated raw and modified membrane.

Thermogravimetric analysis was used to assess the effect of zwitterionic surface modification on the thermal stability of both the commercial CTA membrane and the electrospun nanofibrous membrane (Figure 2a,b). The pristine C1 exhibits two central mass-loss regions. A minimal weight decrease, below roughly 100–120 °C, can be attributed to the removal of physically adsorbed moisture and traces of volatile species trapped in the matrix. The membrane remains intact mainly up to around 230–250 °C, indicating that the commercial CTA structure is thermally stable in the temperature range relevant for FO operation [45,46]. The principal degradation step for C1 occurs between approximately 250 and 400 °C, where a steep decline in mass is observed. This stage corresponds to deacetylation and breakdown of the cellulose triacetate backbone, including scission of ester linkages and subsequent decomposition of the polysaccharide chain. Beyond about 450 °C, only a small fraction of residual char remains, and the curve approaches a plateau close to zero mass, consistent with near-complete decomposition of the organic framework. The zwitterion-modified commercial membrane displays a broadly similar profile, confirming that the underlying CTA matrix is preserved after surface functionalization. A modest additional mass loss is visible in the 150–300 °C region, which is not as pronounced in C1. This extra step is consistent with the thermal degradation of the SBMA/PDA coating layer, which decomposes at lower temperatures than the bulk CTA. Despite this added component, CM1 maintains more than 90% of its initial weight below ~200 °C, and the onset of main backbone degradation remains within the same temperature window as C1. The final residual mass at 700 °C is very close for both samples, indicating that the coating does not contribute to a large inorganic residue (Figure 2a). The behavior of the electrospun membrane differs from that of the commercial CTA membrane because of its distinct polymer composition and the presence of rGO filler. For the Pristine F1, a slight initial mass loss is observed below 100–120 °C, attributed to the desorption of moisture and residual solvent. The membrane retains a high percentage of its original weight up to approximately 250–280 °C, reflecting the intrinsic stability of the PEI-based nanofibrous matrix.

A broad and dominant degradation step occurs between approximately 350 and 600 °C, during which most of the polymer mass is lost. This region corresponds to the scission of the PEI backbone, the breakdown of any PET segments, and the progressive oxidation of organic components, with a small char fraction remaining at the highest temperatures. The presence of rGO and aromatic structures contributes to this residual mass and may slightly shift the degradation profile to higher temperatures compared with a purely polymeric system. For the zwitterion-modified FM1, the TGA curve shows a more complex multi-step pattern. Following the minor moisture loss at low temperatures, a clear additional degradation event is observed, beginning around 170–200 °C and extending to approximately 320–330 °C (Figure 2b). This earlier loss is attributed to the decomposition of the PDA/SBMA coating layer, including the disruption of catechol/amine structures in PDA and the cleavage of the sulfobetaine side groups. Once this coating has decomposed, the curve transitions into a significant second step at higher temperature, corresponding to the degradation of the underlying electrospun matrix [47,48]. Although the onset of substantial mass loss is slightly earlier for FM1 than for F1, the membrane still retains high weight fractions up to temperatures well above those encountered in FO operation. The residual mass at 700 °C for FM1 is comparable to or slightly higher than that of F1, which is consistent with the presence of additional aromatic carbon from PDA and rGO-derived char.

#### 3.1.3. SEM

**Figure 3:**
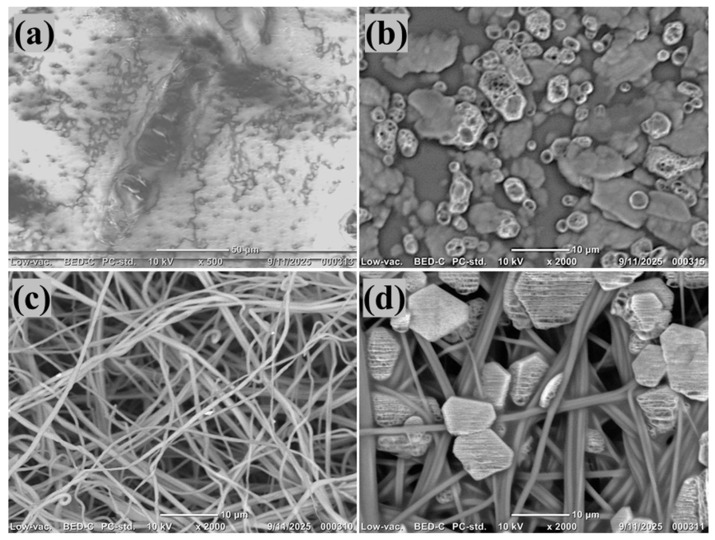
(**a**–**d**) SEM micrographs of C1, CM1, F1, FM1.

The SEM image of the C1 showed a smooth and compact surface with minimal pore characteristics, aligning with its dense support layer structure (Figure 3a). Following zwitterionic modification, the CM1 surface appeared more textured and diverse, featuring granular deposits throughout the membrane, resulting from the effective deposition of PDA and SBMA molecules (Figure 3b). In contrast, the F1 exhibited a consistent fibrous structure with interlinked pores, typical of electrospinning, providing increased surface roughness and porosity (Figure 3b). The SBMA-enhanced FM1, depicted in the accompanying SEM image, displayed fibers covered with unique aggregates and clusters that firmly bond along the nanofibrous network (Figure 3d). These aggregates presumably relate to PDA–SBMA complexes created during the surface polymerization, validating consistent coating throughout the fiber matrix. The alteration resulted in somewhat thicker fibers with decreased visible porosity, indicating the effective grafting of zwitterionic groups [49,50]. These morphological changes are anticipated to greatly improve membrane hydrophilicity and provide antifouling characteristics, rendering FM1 more efficient for FO uses in PW treatment.

#### 3.1.4. XPS

**Figure 4:**
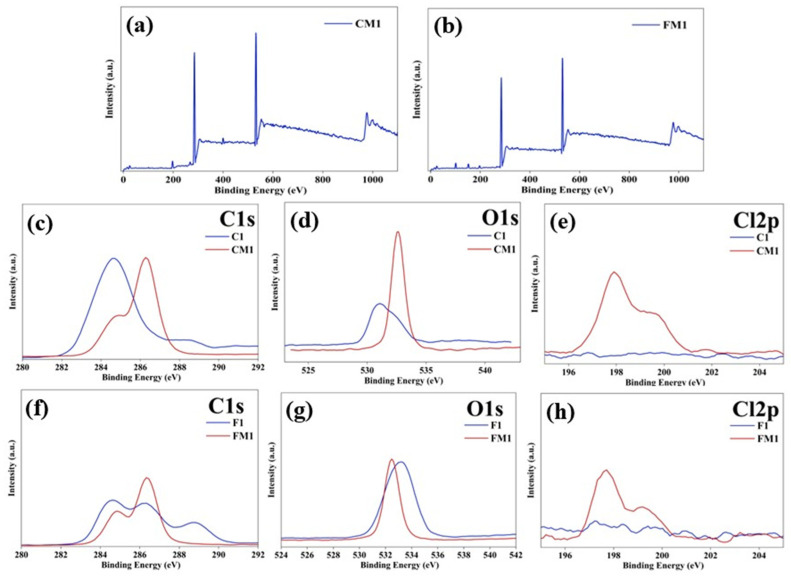
(**a**–**h**) XPS survey spectra for CM1, FM1, and High-resolution (HR) C1s, O1s and Cl2p; XPS spectra for C1, CM1, F1, and FM1.

X-ray photoelectron spectroscopy (XPS) was employed to confirm the successful incorporation of the zwitterionic coating (SBMA/PDA) onto both the C1 and F1. The survey spectrum of CM1 reveals new nitrogen and sulfur features are not present in the pristine C1 membrane. The overall increase in heteroatom signals indicates that the zwitterionic layer forms a uniform surface coverage. The FM1 survey scan shows stronger Cl2p signals compared to CM1, consistent with the higher coating deposition facilitated by the nanofibrous, high-surface-area structure. The electrospun membrane therefore supports thicker or more densely adsorbed PDA/SBMA layers than the CTA membrane (Figure 4a,b).

High-resolution spectra of the C1s, O1s, and Cl2p regions, along with full survey scans, provide insights into the chemical changes introduced by the surface functionalization. In each case, the modified membranes (CM1 and FM1) exhibit distinct shifts in binding energies and changes in peak intensities that correspond to the presence of PDA and SBMA chemical groups. The unmodified commercial membrane (C1) displays the expected C1s contributions typical of cellulose triacetate: a primary peak near ~284.6 eV assigned to C–C/C–H groups, a secondary feature around ~286.5 eV associated with C–O in cellulose rings, and a higher binding-energy component near ~288.5 eV corresponding to O–C=O groups of acetyl esters. After surface modification, the CM1 spectrum shows a noticeable change in both peak shape and relative intensity. The increase in the ~286.0–286.7 eV region reflects contributions from C–N and C–OH functionalities derived from dopamine, while the mild increase toward ~289 eV is consistent with –N^+^(CH_3_)_2_ and –SO_3_^−^ interactions from the SBMA moiety. The overall broadening and amplification in these regions confirm the successful deposition of a zwitterionic layer. The O1s spectrum of C1 is dominated by ester carbonyl oxygen, producing a peak near ~532.5 eV. Following modification, CM1 exhibits an evident shift and intensity increase in the 531–533 eV region. This enhancement is attributed to the following: Phenolic oxygen from PDA’s catechol groups, Hydroxyl groups introduced during dopamine oxidation, Sulfonate oxygen atoms (–SO_3_^−^) from SBMA. These changes reinforce the conclusion that chemically distinct oxygen-containing groups are now present on the membrane surface compared to pristine CTA. The unmodified CTA membrane exhibits nearly no Cl2p signal, consistent with the absence of chlorine-containing functional groups. In contrast, CM1 exhibits a clear Cl2p peak centered around 198–200 eV, which corresponds to the quaternary ammonium–chloride interactions characteristic of sulfobetaine salts. The emergence of this peak provides direct evidence for the presence of SBMA on the surface, as chlorine does not originate from the base CTA membrane (Figure 4c,e).

The pristine electrospun membrane (F1) exhibits multiple carbon-related features due to its polymer blend and the addition of rGO. Peaks near ~284.6 eV (C–C/C–H) and ~285.8–286.5 eV (C–N, C–O) dominate the profile, with a weaker high-binding-energy tail extending toward ~288–289 eV, reflecting carbonyl or imide-related components. After modification, the FM1 spectrum shows an apparent intensification in the C–N/C–OH region (~286–287 eV), reflecting dopamine incorporation. A mild rise in the ~288.5–289 eV zone is also consistent with the zwitterionic SBMA component. Compared to CM1, the FM1 spectrum exhibits more substantial modifications, likely due to the more porous and accessible nanofibrous morphology, which allows deeper penetration and greater interaction between coating components and the fiber surface. The O1s peak of the unmodified electrospun membrane centers near ~532.7 eV, characteristic of imide and ether oxygen in PEI and oxygen-containing groups in rGO. After coating, FM1 exhibits a pronounced increase in peak intensity, accompanied by slight peak broadening. These changes correspond to the introduction of newly formed hydroxyl and catechol oxygen from PDA, Sulfonate oxygen from SBMA, and additional amide or imine related oxygen interactions that occur during dopamine polymerization. This confirms that the zwitterionic coating interacts strongly with the electrospun matrix. Similarly to the commercial membrane, the pristine F1 shows no detectable chlorine signal. The modified FM1 membrane displays a distinct Cl2p contribution centered around 198–200 eV, confirming the presence of quaternary-ammonium-associated chloride ions from SBMA. The higher Cl2p intensity in FM1 relative to CM1 reflects the greater coating uptake due to the high surface area of the nanofibrous substrate (Figure 4f–h).

#### 3.1.5. Contact Angle

**Figure 5:**
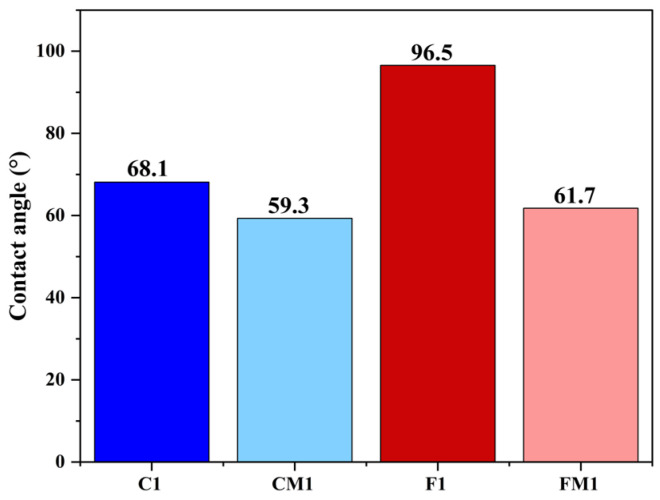
Contact angle measurements of C1, CM1, F1, FM1.

Contact angle measurements were used to evaluate the surface wettability of both commercial CTA and fabricated electrospun membranes before and after zwitterionic modification (Figure 5). C1 exhibited a contact angle of 68.1°, reflecting its moderately hydrophilic character. Following SBMA/PDA surface functionalization, the modified CM1 exhibited a reduced contact angle of 59.3°, indicating improved hydrophilicity due to the zwitterionic coating. In contrast, the electrospun membrane F1 displayed a considerably higher contact angle of 96.5°, consistent with its inherently more hydrophobic nanofibrous surface. After modification, the contact angle of the fabricated membrane decreased significantly to 61.7° for FM1, indicating a substantial improvement in surface wettability, considerably stronger than that measured for the commercial CTA membrane. The intrinsic morphological and chemical characteristics of electrospun substrates can explain this behavior. The pristine F1 possesses a highly porous, rough, and fibrous surface with significant air entrapment within the nanoscale voids. Such hierarchical roughness amplifies hydrophobicity through the Cassie–Baxter wetting regime, resulting in the initially high contact angle of 96.5°. When the surface is coated with the SBMA/PDA layer, two simultaneous mechanisms act to enhance wettability drastically. First, polydopamine forms a conformal adhesive film that smooths the nanofiber surface and reduces air entrapment, shifting the wetting state toward the Wenzel regime where water more easily penetrates surface asperities. Second, the densely grafted SBMA units introduce strongly hydrophilic sulfobetaine groups capable of binding large amounts of structured water through electrostatically driven hydration. The combination of morphological smoothing increased polar functionality, and formation of zwitterion-mediated hydration shells effectively transforms the initially hydrophobic electrospun architecture into a highly water-attractive surface. This synergistic modification accounts for the substantial decrease in contact angle to 61.7° in FM1, a change far more pronounced than in the CTA membrane, whose smoother native surface undergoes a comparatively smaller hydrophobic-to-hydrophilic transition. These reductions in contact angle for both membrane types confirm the effectiveness of the SBMA/PDA layer in promoting surface hydration, suggesting improved antifouling performance and enhanced water–membrane interaction during FO operation.

#### 3.1.6. GC-MS of Produced Water

**Figure 6:**
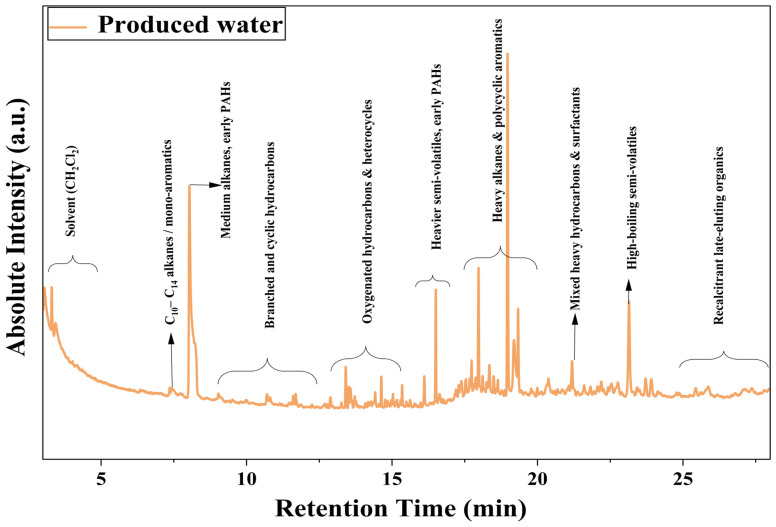
GC-MS Spectra of produced water.

The GC–MS chromatogram of the Permian Basin produced water sample can be seen in Figure 6. PW samples were first extracted into dichloromethane (CH_2_Cl_2_), and the organic phase was used for GC–MS analysis. A few droplets of the reaction mix were dissolved in 2 mL methylene chloride solvent and filtered in a 0.22 nm hydrophobic filter. The filtered solution was stored in a 1.50 mL glass vial for analysis. 1 µL of filtered sample was injected into a Shimadzu QP-2020 GC-MS through an auto-sampler. The equipment includes a DB-5MS capillary column (30 m × 0.25 mm internal diameter) and a packing material diameter of 0.25 µm. The analysis method included a solvent delay of 3.0 min and an electron energy of 70 eV, with an electron ionization mass spectrum in the range of 50–500 (*m*/*z*). Helium (He) was used as carrier gas with a flow rate of 1.0 mL min^−1^. The column temperature program ranged from 50 °C to 180 °C at a heating rate of 15 °C min^−1^, and a holding time of 10 min. All samples were analyzed immediately after preparation to minimize solvent loss and sample degradation.

The plot reveals a chemically heterogeneous mixture characteristic of high-salinity shale-derived waters, with several distinct peak clusters distributed across the entire 0–30 min retention window. The initial portion of the chromatogram (0.5–4 min) could be due to the presence of CH_2_Cl_2_ which is used for the sample preparation. Rapidly declining unresolved complex mixture (UCM) envelope, indicating the presence of light hydrocarbons (C5–C9), volatile aromatics, and condensate-derived compounds typically associated with flowback fluids and entrained gas condensates between 4 and 7 min. As the retention time increases, several sharp, high-intensity peaks appear between 7 and 12 min, corresponding to medium-chain alkanes, branched hydrocarbons, alkyl benzenes, and early eluting polycyclic aromatic hydrocarbons (PAHs). These compounds are commonly observed in reservoir-derived organic fractions and reflect partitioning of crude oil components into the aqueous phase.

The most prominent region of the chromatogram occurs between 15 and 22 min, where multiple high-intensity peaks were observed. As outlined in Table 1, this region is typically associated with long-chain alkanes (C16–C22), alkylated naphthalenes, biphenyl derivatives, and heavier PAHs originating from thermally mature hydrocarbons within the reservoir. Their strong hydrophobicity and structural stability make them especially significant from a membrane-treatment perspective, as these compounds are well known to promote dense organic deposition, pore blockage, and synergistic organic–inorganic fouling during FO operation. Beyond 22 min, the chromatogram transitions into a lower-intensity, but a more complex region containing late-eluting oxidized hydrocarbons, surfactant-derived residues, long-chain acids, and asphaltene-like fragments. Although these species appear in lower abundance, their high molecular weight and surface activity contribute to recalcitrant fouling layers and long-term stability challenges in membrane-based desalination. Overall, the GC–MS analysis confirms that the Permian Basin PW contains a broad suite of volatile, semi-volatile, and high-boiling organic compounds consistent with mature shale formations and high-TDS brines. The distribution of compound classes shown in Table 1 provides a clear link between chemical composition and fouling behavior: lighter fractions contribute to initial adsorption, while heavier alkanes and PAHs dominate long-term organic fouling. These findings help contextualize the observed improvements in flux stability and antifouling performance of the zwitterion-modified membranes, which are better equipped to resist adsorption of hydrophobic and aromatic constituents during FO processing of high-salinity PW.

### 3.2. Forward Osmosis for Produced Water Treatment

#### 3.2.1. Na_3_PO_4_ as Draw Solute

**Figure 7:**
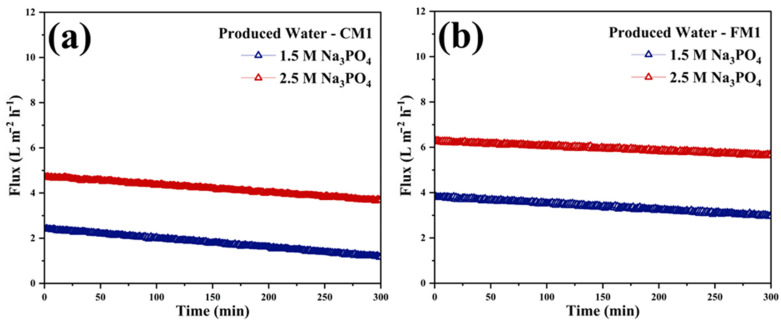
The real-time flux of FO-Produced water (**a**) CM1 and (**b**) FM1 membranes, with Na_3_PO_4_ as draw solute at different concentrations of 1.5 and 2.5 M.

The FO performance under Na_3_PO_4_ draw conditions further highlighted the superiority of the FM1 compared to the CM1 (Figure 7a,b). As shown in the flux profiles, FM1 consistently delivered higher water permeation rates at both 1.5 M and 2.5 M Na_3_PO_4_, with initial fluxes approximately 30–40% greater than those of CM1 and a more stable decline trend throughout the 300 min operation. The improved flux performance of FM1 is attributed to its highly porous electrospun substrate, which minimizes internal concentration polarization, and its uniform SBMA/PDA surface layer, which enhances hydration and mitigates organic and inorganic fouling typically associated with PW matrices. These hydraulic advantages translated directly into improved desalination performance. When operated with 1.5 M Na_3_PO_4_, FM1 achieved a total solids rejection of 33.7%, outperforming CM1, which provided only 26.1% rejection under identical conditions. At 2.5 M Na_3_PO_4_, the disparity became even more pronounced, with FM1 reaching 61.9% TS rejection compared to 49.6% for CM1 (Table 2). The higher rejection capacity of FM1 reflects its enhanced selectivity and reduced reverse solute diffusion, which is supported by its zwitterionic surface chemistry that suppresses solute back-transport and improves the effective osmotic driving force. Overall, the combined trends of higher flux, lower fouling sensitivity, and superior TS removal demonstrate that FM1 provides a more efficient and robust FO platform than CM1 for treating hypersaline PW when phosphate-based draw solutions are utilized.

#### 3.2.2. MgCl_2_ as Draw Solute

**Figure 8:**
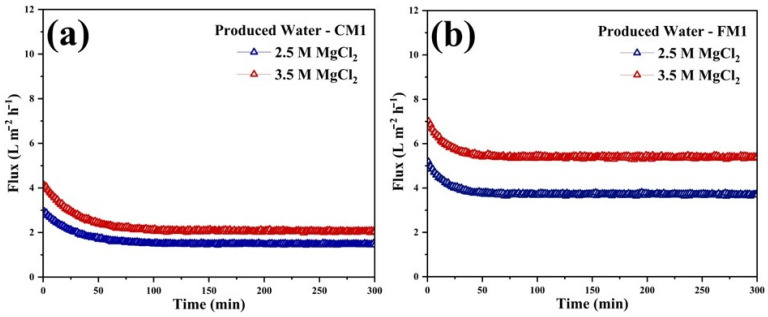
The real-time flux of FO-Produced water (**a**) CM1 and (**b**) FM1 membranes, with MgCl_2_ as draw solute at different concentrations of 1.5 and 2.5 M.

The comparative FO performance of the two zwitterion-modified membranes demonstrated a clear advantage of the zwitterionic modified FM1 over the CM1 when treating high-salinity Permian Basin PW. As shown in the flux profiles, FM1 consistently maintained higher water permeation rates under both 2.5 M and 3.5 M MgCl_2_ draw conditions, with initial fluxes exceeding those of CM1 by approximately 35–40% and stabilizing at higher steady-state values throughout the 5 h operation period (Figure 8a,b). This enhanced flux behavior is attributed to the porous and interconnected nanofibrous architecture of FM1, which reduced internal concentration polarization and promoted more effective transport pathways compared to the denser structure of CM1. The zwitterionic SBMA/PDA coating further strengthened FM1’s advantage by improving surface hydration and minimizing organic deposition, enabling sustained flux with reduced fouling decline. These flux trends were consistent with the desalination performance: FM1 achieved markedly higher total solids removal, reaching 28.8% and 53.3% rejection with 2.5 M and 3.5 M MgCl_2_ draws, respectively, compared to only 16.7% and 46.8% for CM1 under identical test conditions (Table 2). The superior rejection performance of FM1 reflects a combination of its improved selectivity, reduced reverse solute diffusion, and enhanced resistance to fouling by the complex organic–inorganic constituents present in the PW. Collectively, these results confirm that the fabricated zwitterion-modified nanofibrous membrane offers a distinctly more effective FO platform than the modified commercial CTA membrane for the treatment of hypersaline PW.

#### 3.2.3. SEM Micrographs After Forward Osmosis

**Figure 9:**
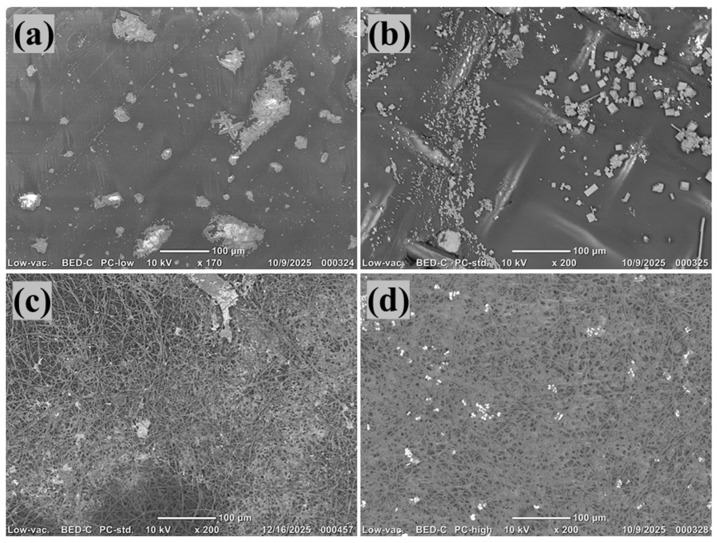
(**a**–**d**) SEM micrographs of CM1-Na_3_PO_4_ as draw solute, CM1-MgCl_2_ as draw solute, FM1-Na_3_PO_4_ as draw solute, and FM1-MgCl_2_ as draw solute.

SEM micrographs acquired after, forward osmosis operation with produced water reveal apparent differences in fouling behavior between the CM1 and the fabricated FM1. As shown in Figure 9a,b, CM1 surfaces exposed to Na_3_PO_4_ at 2.5 M and MgCl_2_ at 3.5 M draw solutions, respectively, exhibit pronounced deposition of heterogeneous foulants, including irregular crystalline aggregates and compact particulate clusters distributed across the membrane surface. The denser and smoother morphology of CM1 appears to promote localized accumulation of inorganic precipitates and organic–inorganic complexes, resulting in partial surface coverage and blockage of transport pathways. In contrast, the FM1 membranes (Figure 9c,d) maintain a largely open and interconnected nanofibrous structure after FO operation, with only sparse and finely dispersed deposits observed. The reduced foulant adhesion on FM1 is attributed to the combined effects of its highly porous electrospun architecture and the zwitterionic SBMA/PDA surface layer, which promotes strong surface hydration and suppresses hydrophobic and electrostatic interactions with produced-water contaminants. Notably, FM1 shows improved fouling resistance under both Na_3_PO_4_ and MgCl_2_ draw conditions, indicating that the zwitterionic coating effectively mitigates deposition of both organic species and salt-derived precipitates.

### 3.3. Mechanism of Wettability, Flux, and Rejection Enhancement

The superior wettability, flux stability, and total solids rejection exhibited by the zwitterion-modified FM1 relative to the CM1 arise from the synergistic coupling of nanofibrous morphology with zwitterionic surface chemistry. The pristine electrospun substrate possesses a highly porous, rough, and fibrous architecture that initially amplifies hydrophobicity through air entrapment within nanoscale voids, characteristic of a Cassie–Baxter wetting regime. Following SBMA/PDA modification, polydopamine conformally coats individual nanofibers, partially smoothing surface asperities and displacing trapped air, while sulfobetaine moieties introduce strongly hydrophilic functionalities capable of forming stable hydration shells through electrostatically driven water binding. This combined morphological and chemical transformation shifts the wetting behavior toward a Wenzel-type regime, resulting in a substantially greater reduction in contact angle for FM1 compared to the smoother CTA-based CM1, which undergoes a more limited surface restructuring. Although SEM images acquired after FO operation indicate a reduction in visible surface porosity due to the coating layer, the underlying nanofibrous network of FM1 remains highly open and interconnected, thereby preserving low mass-transfer resistance and effectively minimizing internal concentration polarization. Concurrently, enhanced surface hydrophilicity reduces interfacial transport resistance and suppresses foulant adhesion, enabling higher and more stable water flux despite partial surface pore narrowing. The improved total solids rejection observed for FM1 further reflects enhanced selectivity governed by both physical and chemical effects, including a reduction in adequate surface pore size and the presence of a hydrated, charge-neutral zwitterionic layer that impedes the diffusion of multivalent salts and organic contaminants through steric and hydration-based exclusion mechanisms. Moreover, SEM observations after produced-water FO operation reveal markedly reduced foulant deposition on FM1 surfaces compared to CM1, consistent with antifouling interactions against hydrophobic organic constituents identified by GC–MS, such as aliphatic hydrocarbons, aromatics, and polycyclic aromatic hydrocarbons. The dense hydration layer formed by SBMA/PDA effectively screens hydrophobic and π–π interactions, limiting the attachment of organic–inorganic foulant complexes involving multivalent ions in hypersaline matrices. Collectively, these mechanisms promote preferential water transport while suppressing solute back-diffusion and fouling accumulation, explaining the consistently higher flux stability and desalination efficiency of FM1 under both MgCl_2_ and Na_3_PO_4_ draw conditions.

## 4. Conclusions

This study provides a comprehensive evaluation of FO treatment of hypersaline Permian Basin PW using a commercial CTA membrane (CM1) and a fabricated electrospun nanofibrous membrane (FM1), each modified with a zwitterionic SBMA/PDA coating. Under both MgCl_2_ and Na_3_PO_4_ draw conditions, FM1 consistently achieved higher water fluxes, slower flux decline, and superior resistance to fouling relative to CM1. The zwitterion-modified electrospun membrane offers a promising pathway toward scalable FO systems capable of treating high-strength PW streams while mitigating fouling and maintaining long-term operational performance. The FO performance under Na_3_PO_4_ draw conditions further highlighted the superiority of the FM1 compared to commercial CM1. As shown in the flux profiles, FM1 consistently delivered higher water permeation rates at both 1.5 M and 2.5 M Na_3_PO_4_, with initial fluxes approximately 30–40% greater than those of CM1 and a more stable decline trend throughout the 300 min operation. The improved flux performance of FM1 is attributed to its highly porous electrospun substrate, which minimizes internal concentration polarization, and its uniform SBMA/PDA surface layer, which enhances hydration and mitigates organic and inorganic fouling typically associated with PW matrices. These hydraulic advantages translated directly into improved desalination performance. When operated with 1.5 M Na_3_PO_4_, FM1 achieved a total solids rejection of 33.7%, outperforming CM1, which provided only 26.1% rejection under identical conditions. At 2.5 M Na_3_PO_4_, the disparity became even more pronounced, with FM1 reaching 61.9% TS rejection compared to 49.6% for CM1. The higher rejection capacity of FM1 reflects its enhanced selectivity and reduced reverse solute diffusion, which is supported by its zwitterionic surface chemistry that suppresses solute back-transport and improves the effective osmotic driving force. Overall, the combined trends of higher flux, lower fouling sensitivity, and superior TS removal demonstrate that FM1 provides a more efficient and robust FO platform than CM1 for treating hypersaline PW when phosphate-based draw solutions are utilized.

## Figures and Tables

**Table 1:** GC-MS peak assignment table for Permian basin-produced water.

Retention Time (min)	Relative Intensity (a.u.)	Compound Class	Specific Identifications	Typical Origin in PW
**4.0–5.5**	Low	Branched and cyclic C7–C9 hydrocarbons	Trimethylpentane isomers, ethylbenzene	Drilling additives, condensate
**5.5–7.5**	Low	C10–C14 alkanes & mono-aromatics	2-Propenoic acid, 2-methyl-, 2-methylpropyl ester	Oil components, surfactant breakdown
**7.5–9.5**	Medium	Medium-chain alkanes, early PAHs	2-Propenoic acid, 2-methyl-, 2-methylpropyl ester	Reservoir hydrocarbons
**9.5–12**	Medium–High	Branched alkanes, alkylcycloalkanes	5-Cyclopropylcarbonyloxypentadecane	Separator carryover
**12–15**	Medium	Oxygenated hydrocarbons & heterocycles	2,2-Dimethyl-1-propyl methylphosphonofluoridate, 9-methylheptadecane	Oxidation products, inhibitors
**15–18**	High	Heavier semi-volatiles, early PAHs	Octadecane, 1-isocyanato-, Tetradec, 2,4-Di-tert-butylphenol, Octadec	Mature reservoir oil
**18–21**	Very High	Heavy alkanes & polycyclic aromatics	Heneicosane, Octadecane, 1-isocyanato-	Thermally stable crude fractions
**21–24**	Medium	Mixed heavy hydrocarbons & surfactants	Nonahexacontanoic acid, Octadecane, 1-isocyanato-	Production chemicals
**24–27**	Low	High-boiling semi-volatiles	Trichloroacetic acid, decyl ester	Oxidized oil fractions
**27–30**	Low	Very heavy, late-eluting organics	Nonahexacontanoic acid	Deep reservoir signatures

**Table 2:** Total Solid rejection of Produced water using CM1 and FM1 as well as Na_3_PO_4_ and MgCl_2_ as draw solutions at two different concentrations.

Membrane	Draw Solution (M)	%TS Rejection
CM1	Na_3_PO_4_—1.5 M	26.1%
FM1		33.7%
CM1	Na_3_PO_4_—2.5 M	49.6%
FM1		61.9%
CM1	MgCl_2_—2.5 M	16.7%
FM1		28.8%
CM1	MgCl_2_—3.5 M	46.8%
FM1		53.3%

## Data Availability

The original contributions presented in this study are included in the article. Further inquiries can be directed to the corresponding author.
